# What happens after treatment? A systematic review of relapse, remission, and recovery in anorexia nervosa

**DOI:** 10.1186/s40337-017-0145-3

**Published:** 2017-06-14

**Authors:** Sahib S. Khalsa, Larissa C. Portnoff, Danyale McCurdy-McKinnon, Jamie D. Feusner

**Affiliations:** 1 Laureate Institute for Brain Research, 6655 S Yale Ave, Tulsa, OK 74136 USA; 2 Oxley College of Health Sciences, The University of Tulsa, 1215 South Boulder Ave W, Tulsa, OK 74119 USA; 3 Department of Clinical Psychology, Teachers College, Columbia University, 525 W 120th St, New York, NY 10027 USA; 4 Department of Pediatrics, The University of California Los Angeles, 757 Westwood Plaza, Los Angeles, CA 90095 USA; 5 Department of Psychiatry and Biobehavioral Sciences, The University of California Los Angeles, Semel Institute of Neuroscience and Human Behavior, 760 Westwood Plaza, Los Angeles, CA 90024 USA

**Keywords:** Anorexia nervosa, Treatment, Outcome, Relapse, Remission, Recovery, Prevention, Eating disorder, Bulimia nervosa

## Abstract

**Background:**

Relapse after treatment for anorexia nervosa (AN) is a significant clinical problem. Given the level of chronicity, morbidity, and mortality experienced by this population, it is imperative to understand the driving forces behind apparently high relapse rates. However, there is a lack of consensus in the field on an operational definition of relapse, which hinders precise and reliable estimates of the severity of this issue. The primary goal of this paper was to review prior studies of AN addressing definitions of relapse, as well as relapse rates.

**Methods:**

Data sources included PubMed and PsychINFO through March 19th, 2016. A systematic review was performed following the PRISMA guidelines. A total of (*N* = 27) peer-reviewed English language studies addressing relapse, remission, and recovery in AN were included.

**Results:**

Definitions of relapse in AN as well as definitions of remission or recovery, on which relapse is predicated, varied substantially in the literature. Reported relapse rates ranged between 9 and 52%, and tended to increase with increasing duration of follow-up. There was consensus that risk for relapse in persons with AN is especially high within the first year following treatment.

**Discussion:**

Standardized definitions of relapse, as well as remission and recovery, are needed in AN to accelerate clinical and research progress. This should improve the ability of future longitudinal studies to identify clinical, demographic, and biological characteristics in AN that predict relapse versus resilience, and to comparatively evaluate relapse prevention strategies. We propose standardized criteria for relapse, remission, and recovery, for further consideration.

## Plain English Summary

Relapse occurs frequently in individuals receiving treatment for anorexia nervosa. However, there is no common agreement on how to define relapse. In this study, we reviewed previous studies of relapse, remission, and recovery following treatment for anorexia nervosa. We found that there were many different definitions for these terms, which resulted in different estimates of relapse rate. To understand what drives relapse it is important to have a consistent definition across studies. To help this discussion we propose common criteria for relapse, remission, and recovery from anorexia nervosa.

## Background

Anorexia nervosa (AN) is a serious psychiatric illness with amongst the highest mortality rates of any mental disorder—up to 18% in long-term follow-up studies [1–3]. Most cases emerge during adolescence, and tend towards a protracted and chronic course [4, 5]. In females, AN has a point prevalence of 0.3–1.0% and lifetime prevalence of 1.2–2.2% [6]. Treatment often succeeds in temporarily restoring weight, but AN individuals are at an exceedingly high risk for early relapse [7], and upwards of 50% relapse within the first year after successful hospital treatment [8]. The current lack of robust and reliable responses to treatment highlights the need for an improved ability to predict illness trajectories.

The primary focus of this review is on how relapse is defined following treatment for AN. Since relapse is typically defined relative to recovery and remission, we also consider how recovery and remission are defined. Pike has previously eloquently reviewed relapse, recovery, remission, and response in AN [8]. However, since then 11 studies have addressed this topic. The current review therefore incorporates these additional publications.

In preparing this review, a lack of clarity and uniformity with regard to how to best define relapse, recovery, and remission was apparent. This perspective is reinforced by a literature review of remission in eating disorders concluding that the definitions and associated rates vary considerably [9]. Fifteen years ago, a European collaboration of experts (COST Action B6) adapted definitions for relapse, recovery, partial and full remission, and recurrence from the depression literature to AN and bulimia nervosa (BN) [10]. Despite rigorous consensus-building and empirical testing of 233 inpatients with AN, these criteria have not been uniformly adopted by the field. To date there are no consensus guidelines available for clinicians or researchers at the professional or institutional level providing standardized operational definitions of relapse, recovery, or remission in AN. This is limiting. A greater consensus regarding the definition of these constructs would be of considerable benefit to clinicians, researchers, patients, and family members, by allowing all constituents to speak the same language.

We performed a focused review of the extant literature with the primary aim of examining how these terms have been defined, in order to improve definitions of relapse, recovery and remission in AN. Reviewing relapse rates was a secondary goal. We propose a set of standardized criteria for relapse, recovery, and remission from AN, which are internally cohesive and can facilitate longitudinal assessment by clinicians and researchers.

## Methods

### Search and study selection

We conducted a systematic qualitative review according to the PRISMA guidelines, searching the PubMed and PsychINFO databases. We used keywords for either “anorexia nervosa” or “eating disorders” along with “relapse,” or “recovery,” or “remission.” We used an open search procedure. We also performed the same searches on Google Scholar to locate relevant articles that the other search methods possibly overlooked (none were identified). ﻿Our search covered articles that were ﻿published from 1975 to March 19th, 2016﻿. Titles and abstracts were evaluated and full text was reviewed for relevant studies. References sections were screened manually for additional studies unidentified via database search. 

### Eligibility criteria

Participants had to meet ICD-10, DSM-III, IV, or 5 diagnostic criteria for AN for inclusion. Studies (*n* = 1) focusing on binge eating providing relevant information regarding relapse risk in AN or treatment outcomes of AN were also included. Studies examining BN and AN were included, but not those focused solely on BN (*n* = 2) (except for one [11] that provided treatment information pertinent to AN binge-purge (AN-BP) subtype). Omitted studies included those focused on unspecified eating disorders (*n* = 2), comorbid psychiatric disorders (*n* = 2), or those without clinical descriptions of relapse or recovery (*n* = 3). Non-English language articles were excluded (*n* = 6). 

### Data review and study quality assessment

Three authors (LCP, SSK, and JF) independently extracted the following data from the selected studies: first author, publication year, country, and whether the study was related to relapse, recovery, or remission. To evaluate the quality of the studies, we performed a systematic review of each article using the National Heart, Lung, and Blood Institute Study Quality Assessment Tool [12]. This tool provides a rating checklist for each study type. Three authors (LCP, DM, SSK) independently evaluated each study according to the rating checklist, and rendered a rating of “Good” or “Fair” or “Poor.” Study quality was determined by comparing ratings agreement, with consensus required among reviewers. Discrepancies in study quality rating were reconciled via discussion of the individual items on the ratings checklist to arrive at consensus agreement on the quality indicator. Disagreements were resolved through discussion and consensus. There were no biases or poor methods identified that warranted exclusion from the review.

## Results

**Fig. 1:**
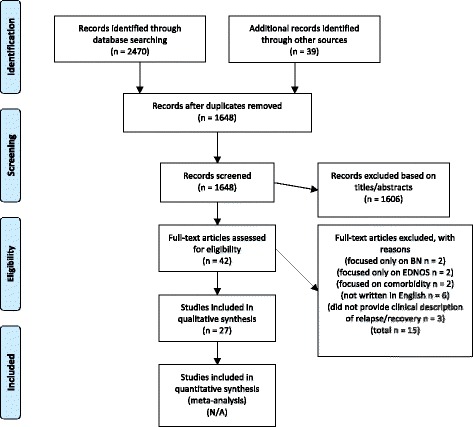
Prisma diagram

We identified 27 studies meeting eligibility criteria (see Fig. 1). ﻿ ﻿An overview of pertinent study characteristics and definitions of recovery/remission and relapse in AN are listed in Tables 1 and 2.﻿ Definitions of relapse were fundamental to understanding the reported rates in these studies. Our review revealed widely varied definitions of relapse and recovery/remission in AN. Definitions of recovery and remission are reviewed first since relapse is predicated upon them.

**Table 1:** Definitions of recovery and remission, according to individual studies identified by the literature search

Authors	Criteria	Duration	Study quality
Definitions of Recovery			
Martin, 1985 [28]	“Excellent”: > 90% of their ideal weight, regular menstrual patterns, and eating and social patterns were normal	Not specified	Fair
Norring and Sohlberg, 1993 [34]	“Well” defined as having no eating disorder diagnosis or remnants of the weight and/or shape preoccupation	Not specified	Good
Eckert et al., 1995 [29]	≥85% of ideal body weight, cyclical menses, and no significant disturbance in eating or weight control behavior or body image disturbance	Not specified	Good
Strober et al., 1997 [4]	Free of all criterion symptoms of anorexia nervosa or bulimia nervosa	8 weeks	Good
Fichter and Quadflieg, 1999 [21]	Outcome “good” defined using Morgan-Russell criteria	Not specified	Fair
Pike, 1998 [8]	≥90% of ideal body weight or BMI ≥20, resumption of menses, absence of binge eating or compensatory behaviors, Eating Disorder Examination subscales within 2 SD of normal	8 weeks	Fair
Herzog, et al., 1999 [32]	Absence of all symptoms or 1–2 residual symptoms—Psychiatric Status Rating (PRS) score of 1 or 2	8 weeks	Good
Lowe et al., 2001 [22]	Outcome “good” defined using Morgan-Russell and PSR 1	Not specified	Good
Kordy et al., 2002 [10]	AN-R: BMI > 19, no extreme fear of weight gain AN-BP: BMI > 19, no extreme fear of weight gain, no vomiting or laxative abuse, no binges	12 months	Good
Carter et al., 2004 [15]	BMI above 20	Not specified	Good
Walsh et al., 2006 [14]	BMI above 19	No information	Good
Eisler et al., 2007 [20]	Outcome “good” defined using Morgan-Russell criteria	Not specified	Good
Bodell and Mayer, 2011 [24]	No DSM–IV criteria of AN	8 weeks	Fair
Bardone-Cone et al., 2010 [30]	Full recovery: BMI ≥ 18.5, absence of binge-eating, purging or fasting for at least 3 months, not meeting criteria for current eating disorder, all EDE-Q subscales within 1 SD of normal Partial recovery: same as above, but not needing to satisfy EDE-Q criterion	Not specified	Good
Carter et al., 2012 [7]	BMI of 20 and reported no more than one BP episode before the end of treatment.	2 weeks BMI and no BP behaviors over the previous 28 days at the end of treatment	Good
Definitions of Full Remission			
Morgan and Hayward, 1988 [23]	≥85% of ideal body weight, regular menses, and no binge eating or purging behaviors	Not specified	Fair ^a^
Pike, 1998 [8]	≥90% of ideal body weight or BMI ≥20, resumption of menses, absence of binge eating or compensatory behaviors, EDE subscales within 2 SD of normal	Not specified	Fair
Stice et al., 2000 [27]	BMI ≥17.5, regular menses, and no current subthreshold or full threshold eating disorder	Not specified	Good ^a^
Kordy et al., 2002 [10]	AN-R: BMI > 19, no extreme fear of weight gain AN-BP: BMI > 19, no extreme fear of weight gain, no vomiting or laxative abuse, no binges	12 weeks	Good
Keel et al., 2005 [17]	Absence of all symptoms or 1–2 residual symptoms—PSR score ≤2	8 weeks	Good
Clausen, 2008 [18]	PSR score ≤2	12 weeks	Good
Helverskov et al., 2010 [16]	Absence of all symptoms/1–2 Residual symptoms—PSR score of 1 or 2	12 weeks	Good
Definitions of Partial Remission			
Lowe et al., 2001 [22]	Outcome “improved” defined using Morgan-Russell criteria and PSR 2, 3, or 4	Not specified	Good
Kordy et al., 2002 [10]	AN-R: BMI > 17.5 AN-BP: BMI > 17.5 in addition to ≤1 binge per week and no vomiting or laxative abuse	4 weeks	Good
Clausen, 2008 [18]	PSR score ≤3	12 weeks	Good
Helverskov et al., 2010 [16]	PSR score of 3	12 weeks	Good

^a^ No NHLBI systematic criteria available to rate this study type; quality rating reflects consensus agreement between two rater assessments

**Table 2:** Definitions of relapse, according to individual studies identified by the literature search

Authors	Criteria	Duration	Study quality
Definitions of Relapse			
Isager et al., 1985 [33]	Loss of ≥15% of weight acquired during course of treatment (if resulting in weight ≤50 kg)	Any point in time within a 1 year period	Good
Martin, 1985 [28]	If the patient required further psychiatric treatment after discharge during follow–up period	Not specified	Fair
Norring and Sohlberg, 1993 [34]	“Ill” defined as having an eating disorder	Not specified	Good
Eckert et al., 1995 [29]	Loss of ≥15% of average body weight (based on Metropolitan Height-Weight Chart, 1959), after achieving normal body weight	Any point after achieving normal weight during inpatient treatment or the follow up period	Good
Strober et al., 1997 [4]	Full (“syndromal”) relapse: weight <85% of ideal body weight and recurrence of psychological symptoms Partial (“subsyndromal”) relapse: recurrence of psychological symptoms but ≥85% of ideal body weight	Not specified	Good
Fichter and Quadflieg, 1999 [21]	Outcome “poor” defined using Morgan-Russell criteria	Not specified	Fair
Pike 1998 [8]	BMI ≤ 18.5 or weight ≤85% of ideal body weight; a minimum 1 SD increase on the Eating Disorder Evaluation; loss of menstrual functioning if it has been previously normal; increase in restriction leading to weight loss; and possibly increased binge eating, compensatory behavior, or associated medical problems	Not specified	Fair
Herzog, et al., 1999 [32]	Return to full criteria symptoms and/or Psychiatric Status Rating (PSR) score of 5 or 6	8 weeks following a state of full recovery	Good
Lowe et al., 2001 [22]	Outcome “poor” defined using Morgan-Russell criteria and PSR score of 5 or 6	Not specified	Good
Kordy et al., 2002 [10]	Change from partial or full remission to full syndrome according to DSM-IV	Not specified	Good
Carter, et al., 2004 [15]	BMI below 17.5 and/or at least one episode of binge eating/purging behavior per week	3 consecutive months	Good
Keel, et al., 2005 [17]	Return to full criteria symptoms and/or PSR score of 5 or 6	Not specified	Good
Walsh et al., 2006 [14]	BMI below 16.5 for 2 consecutive weeks, or severe medical complications, or risk of suicide, or development of another psychiatric disorder requiring treatment	2 consecutive weeks (low BMI)	Good
Eisler et al., 2007 [20]	Outcome “poor” defined using Morgan-Russell criteria	Not specified	Good
Clausen, 2008 [18]	PSR score ≥3	3 months	Good
Bodell and Mayer, 2011 [24]	Poor outcome, BMI ≤18.5 (using modified Morgan-Russell criteria)	Not specified	Fair
Helverskov, et al., 2010 [16]	Return to full criteria symptoms and/or PSR score of 5 or 6	Not specified	Good
Carter et al., 2012 [7]	BMI < 17.5 or at least one episode of binge eating/purging behavior per week	3 consecutive months	Good
McFarlane et al., 2015 [31]	AN: BMI < 18.5 AN-BP: average 4 episodes of bingeing and/or vomiting per month, or BMI < 18.5	3 consecutive months	Good

### Definitions of recovery and remission

Recovery typically requires an extended period of time during which minimal or no criteria for the disorder are met, whereas remission requires a shorter duration [13]. The literature can roughly be divided into articles that (1) define remission/recovery based solely on weight measurement, (2) define remission/recovery based solely on symptom reports, (3) define remission/recovery based solely on weight and symptom reports, i.e., diagnostic criteria available at the time. We briefly review these studies next (Table 1 lists studies providing definitions of partial remission, full remission, and recovery).

Several studies used body mass index (BMI) as the only criterion for recovery. Cutoffs included a BMI above 19 [14] or 20 [7, 15]. In contrast, some described remission based solely on psychiatric symptoms. In one, full remission was defined as an absence of all symptoms or only “residual symptoms” for at least 12 weeks, and partial remission was defined as a reduction of symptoms to a sub-diagnostic level for at least 12 weeks [16]. Adopted from the MacArthur guidelines for depression [13], Keel et al. [17] defined full remission as a Psychiatric Status Rating (PSR) score of ≤2 for 8 weeks. Clausen [18] used the same score for 12 weeks, and defined partial remission as a PSR ≤3 for 12 weeks.

Other articles described outcomes in terms of body weight and menstruation, using terminology such as “good,” “intermediate,” “poor,” or “died” [19–22]. These criteria, or modifications of them, are often referred to as the “Morgan-Russell” criteria [19]. A later version specified remission as weight ≥85% of ideal body weight, regular menses, and no bingeing or purging behaviors [23]. Modifying these criteria, recovery was later defined as not meeting AN DSM-IV-TR criteria for a minimum of 8 weeks [24].

Several proposed definitions included both weight and clinical symptoms. Pike [8] defined remission as ≥90% of ideal body weight, resumption of menses, absence of compensatory behaviors, and Eating Disorder Examination (EDE) [25] subscales within 2 standard deviations (SD) of normal. Recovery was defined as meeting remission criteria for at least 8 weeks. Strober et al. [4] defined full recovery as the absence of all criteria for at least 8 weeks, and partial recovery as a “good outcome” (weight within 15% of average and normal menstruation) from the Morgan-Russell criteria [19]. Other studies did not have a duration criterion for the absence of symptoms but used the “good outcome” criteria to define recovery [20–22]. Stice’s Eating Disorder Diagnostic Scale defined remission as BMI ≥17.5, regular menses, and no subthreshold or full threshold eating disorder [26, 27]. Martin [28] defined recovered as having a global rating scale of “excellent,” meaning an individual was >90% ideal weight, had regular menstruation, and normal eating and social patterns. Eckert et al. [29] defined “recovered” as within 15% of ideal body weight, cyclical menses, and no significant disturbance in eating or weight control behaviors or body image disturbance. Kordy et al. [10] defined full recovery for restricting AN as a BMI >19 and no extreme fear of weight gain for 12 months (plus no purging and no binges for 12 months for AN-BP). They defined full remission for both subtypes as meeting the same criteria for 3 months. Partial remission was a BMI >17.5 and ≤1 binge per week and no vomiting or laxative abuse for 1 month in AN-BP. Another proposed definition of full recovery was a BMI ≥18.5, absence of binging, purging, or fasting for at least 3 months, not meeting criteria for a current eating disorder, and all EDE-Questionnaire (EDE-Q) subscales within 1 SD of normal [30]. They defined partial recovery as the same without the EDE-Q criterion.

### Definitions of relapse

Different definitions of relapse were identified (see Table 2). Some definitions were dependent on weight or BMI measures including: BMI < 16.5 for 2 weeks [14], and BMI < 17.5 [7, 15] or <18.5 [31] for three consecutive months. Other definitions included 15% loss of average body weight after achieving normal body weight, either during the index hospitalization or any time during the 10-year follow-up period [29]. Strober et al. [4] similarly defined relapse as <85% ideal body weight, which could occur post-discharge or post-recovery. Furthermore, relapse could be partial if the individual had recurrence of psychological symptoms but sustained 85% of ideal weight, or full relapse if both psychological symptoms returned and body weight dropped to less than 85%. Several groups [19–22, 24] defined relapse as Morgan-Russell criteria of “poor” (BMI ≤18.5).

Other definitions of relapse were dependent on psychiatric symptoms or a combination of psychiatric symptoms and weight changes. Kordy et al. [10] used a definition of change from DSM-IV partial or full remission to full syndrome. Clausen [18] defined relapse as PSR ≥ 3 or PSR ≤ 2 after 3 months remission. Relapse has also been defined as meeting full syndrome criteria (PSR ≥ 5) after 8 weeks of remission [17, 32] and after 12 weeks of remission [16]. Pike’s [8] more in-depth definition of relapse includes weight loss, EDE increase, medical issues, and a return of disordered eating, whereas Martin’s [28] is the simplest, requiring only that an individual needs psychiatric intervention.

### Rates of Relapse

Relapse rates of AN were highly variable ranging from a low of 9% to a high of 52% following treatment, with the majority of studies reporting rates greater than 25% [4, 7, 10, 14–18, 21, 22, 24, 28, 29, 32–34]. Studies suggest that adolescents [4, 20, 28] and individuals with restricting subtype AN [7, 29] have a lower likelihood of relapse. The first year is the most critical, with particular risk of relapse occurring as early as 3 months post-treatment [4, 7, 15, 32]. Not surprisingly, those who recover fully have lower relapse rates (9%) than those who only partially recover (35%) [10]. Together, these results suggest that while most patients experience brief episodes of recovery, a large proportion relapse. Moreover, the risk is particularly high within the first year.

### Follow-Up Variability

There was substantial variability in the literature for follow-up procedures. Initial evaluation time points ranged from 4 weeks to 17 months post-treatment [4, 7, 14, 15, 17, 20, 28, 32, 35]. Some studies utilized only a single follow-up time point [15, 28], whereas others followed patients across multiple time points [4, 7, 14, 17, 20, 32, 35]. Some studies had regular follow-up visits (e.g., every 4 weeks [14], 3 months [7]), whereas others had irregularly spaced follow-ups (e.g., 2, 6 and 12 year follow up [35]).

Variable follow-up intervals could complicate estimations of relapse rates, since relapse rates can vary by duration of the study follow-up. According to this view, shorter follow-up durations might be associated with lower relapse rates than longer durations. We identified articles supporting this possibility. For example, relapse in a study measuring at 6 months was lower (9% for fully recovered and 35% for partially recovered) [10] versus studies measuring at 1-year (27–70%) [7, 14] (see Table 3). Relapse rates also varied by remission criteria, with stricter remission criteria displaying lower relapse rates than less stringent criteria. This is evidenced by two 10-year longitudinal studies. Eckert and colleagues [29] reported higher relapse rates (42%) with less stringent relapse criteria and Strober and colleagues [4] reported lower relapse rates (29.5%) with stricter relapse criteria.

**Table 3:** Rates of relapse identified by the literature search^a^

Authors	Definition	Rate	Sample size	Follow up rate	Study quality
Rates of Relapse					
Isager et al., 1985 [33]	Loss of ≥15% of weight acquired during course of treatment (if resulting in weight ≤50 kg) any point in time within a 1 year period	26% AN	151	Not reported at 4–22 years (mean follow up: 12.5 years)	Good
Norring and Sohlberg, 1993 [34]	“Ill” defined as having an eating disorder or dead	25% AN	48	62% at 6 years	Good
Eckert et al., 1995 [29]	Loss of ≥15% of average body weight (based on Metropolitan Height-Weight Chart, 1959), after achieving normal body weight	42% AN	76	100% at 10 years	Good
Herzog et al., 1999 [32]	Relapse full criteria of symptoms or PSR score of 5 or 6 for 8 weeks following a state of recovery	40% AN	136	93% at 7.5 years	Good
Kordy et al., 2002 [10]	Change from partial or full remission to full syndrome according to DSM-IV	9% AN who were in full remission/recovery; 35% AN who were in partial remission	233	67% at 2.5 years	Good
Carter et al., 2004 [15]	BMI below 17.5 for 3 consecutive months or at least one episode of binge eating/purging behavior per week for 3 consecutive months following a state of recovery	35% AN	51	100% at 0.5 years (mean follow up: 1.3 ± 0.4 years)	Good
Carter et al., 2012 [7]	BMI below 17.5 for 3 consecutive months or at least one episode of binge eating/purging behavior per week for 3 consecutive months following a state of recovery	41% AN overall; 70% AN–BP, 30% AN–R	100	Not reported at 1 year	Good
Walsh et al., 2006 [14]	BMI below 16.5 for 2 consecutive weeks, or severe medical complications, or risk of suicide, or development of another psychiatric disorder requiring treatment	27% of the placebo group and 29% of the fluoxetine group	89 (44 placebo group; 49 fluoxetine group)	43% at 1 year	Good
Keel et al., 2005 [17]	Full criteria symptoms/PSR score of 5 or 6	36% AN group	136	93% at 9 years	Good
Helverskov et al., 2010 [16]	Full criteria symptoms/PSR score of 5 or 6	19% AN: full or partial relapse	58	50% at 2.5 years	Good
Martin, 1985 [28]	“Excellent”: > 90% of their ideal weight, regular menstrual patterns, and eating and social patterns were normal. “Good”: some but not incapacitating difficulty in one of these areas only	9% of AN adolescents	25	100% at 5 years	Fair
Strober et al., 1997 [4]	Weight falls below 85% of total body weight/recurrence of psychological symptoms	29.5% AN post-discharge relapse; 9.8% AN post-partial-recovery relapse	95	87% at 0.5 years Final follow up at 15 years	Good
Fichter and Quadflieg, 1999 [21]	Outcome “poor” defined using Morgan-Russell criteria	20.8% AN	103	98% at 6 years	Good
Lowe et al., 2001 [22]	Outcome “poor” defined using Morgan-Russell criteria and PSR score of 5 or 6	26% AN	63	90% at 21 years	Good
Eisler et al., 2007 [20]	Outcome “poor” defined using Morgan-Russell criteria	34.2% AN adolescents	38	95% at 5 years	Good
Clausen, 2008 [18]	PSR score ≥ 3	8.6%	51	69% at 2.5 years	Good
Bodell and Mayer, 2011 [24]	Poor outcome BMI less than 18 (using Modified Morgan Russell criteria)	52% of AN group poor outcome	21	86% at one year	Fair

^a^ *N* = 1474 patients included across all studies. Reported follow up rates varied tremendously, from 43 to 100% across 0.5 to 21 years. However, the average follow up rate was relatively high at 82.2%

## Discussion

The main finding of this review is that there are almost as many definitions of relapse, remission, and recovery as there are studies of them. To help rectify this state of affairs, we suggest that the eating disorders research and clinical communities evaluate, test, and ultimately adopt standardized definitions for relapse, remission, and recovery. Depression [13], bipolar disorder [36], and schizophrenia [37] researchers already utilize standardized definitions of these constructs. Consensus guidelines for response, partial response, remission, recovery, and relapse in obsessive compulsive disorder were also recently proposed [38]. However, we could identify no such definitions for AN across organizational websites, including: the Academy for Eating Disorders, Eating Disorders Research Society, National Eating Disorders Association, and the European Council on Eating Disorders.

Standardizing how relapse and recovery are defined in research could substantially improve our understanding of the pathophysiology of AN and help ground studies of efficacy and effectiveness, as argued previously [39, 40]. Consensus would increase the quality of meta-analytic studies. It would facilitate multi-site comparisons, which are necessary to improve statistical power for studying this relatively rare condition. Precise and consistent terminology would also enhance communication amongst researchers, clinicians, and caregivers.

**Fig. 2:**
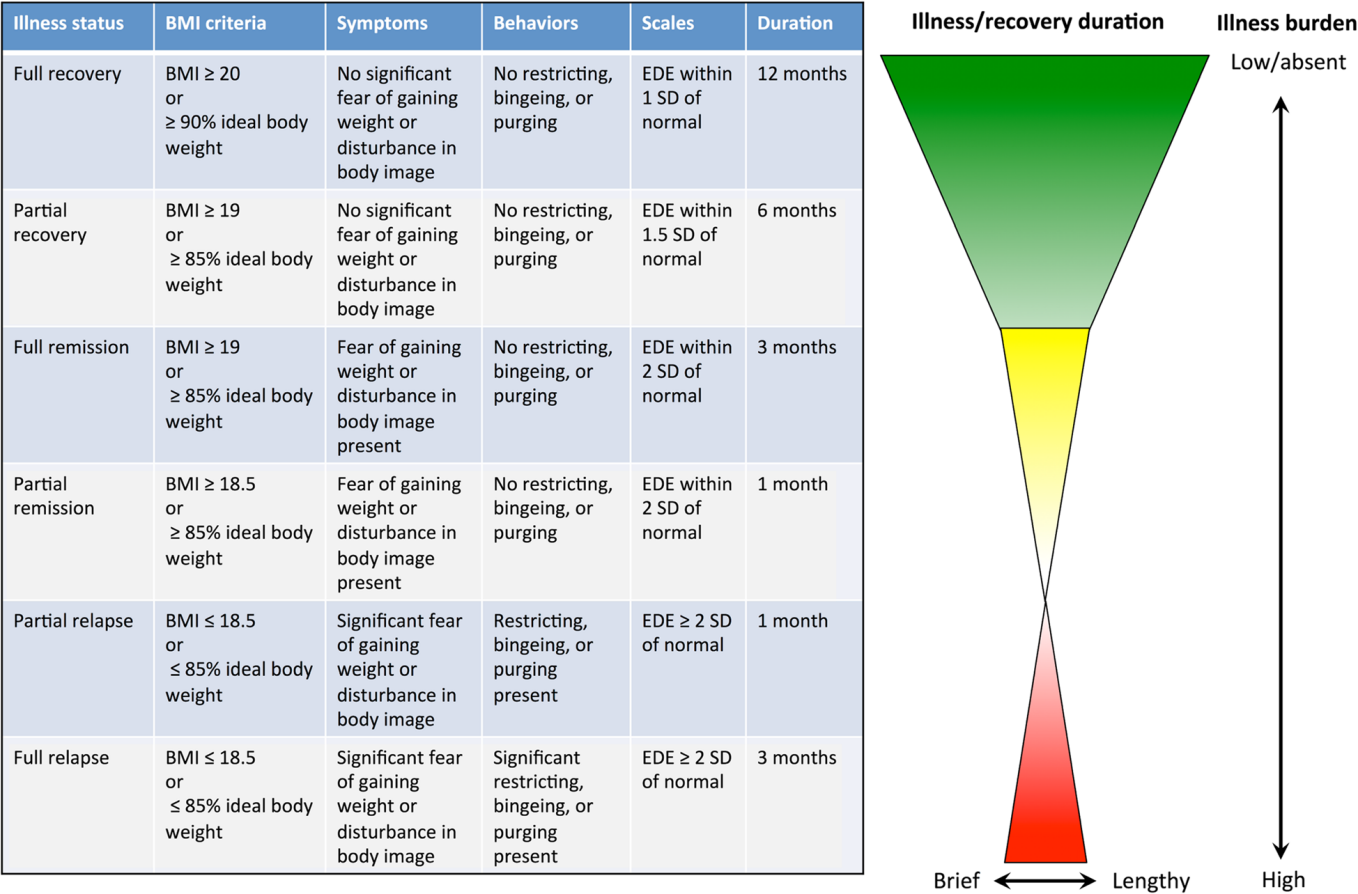
Proposed standardized definitions of relapse, remission, and recovery. These standardized definitions were synthesized from the different criteria for relapse, remission, and recovery in individual studies identified by our systematic review. We include a graphical representation of these definitions as a useful heuristic tool for conceptualizing the major transition points (relapse in red, remission in yellow, recovery in green) while at the same time underscoring the continuum of pathology existing within each stage. Note 1: since weight and height normally increase until age 20 in pediatric and adolescent populations, age- and gender- adjusted BMI percentiles for determining expected body weight (EBW) are more appropriate in these subgroups, as demonstrated by [52]. Note 2: determination of ideal body weight is complex, and subject to consideration of racial, ethnic, demographic, and cultural factors [53]. Note 3: Symptoms and behaviors are discrete variables, which are rated/ascertained by the clinician based on all available clinical information

**Fig. 3:**
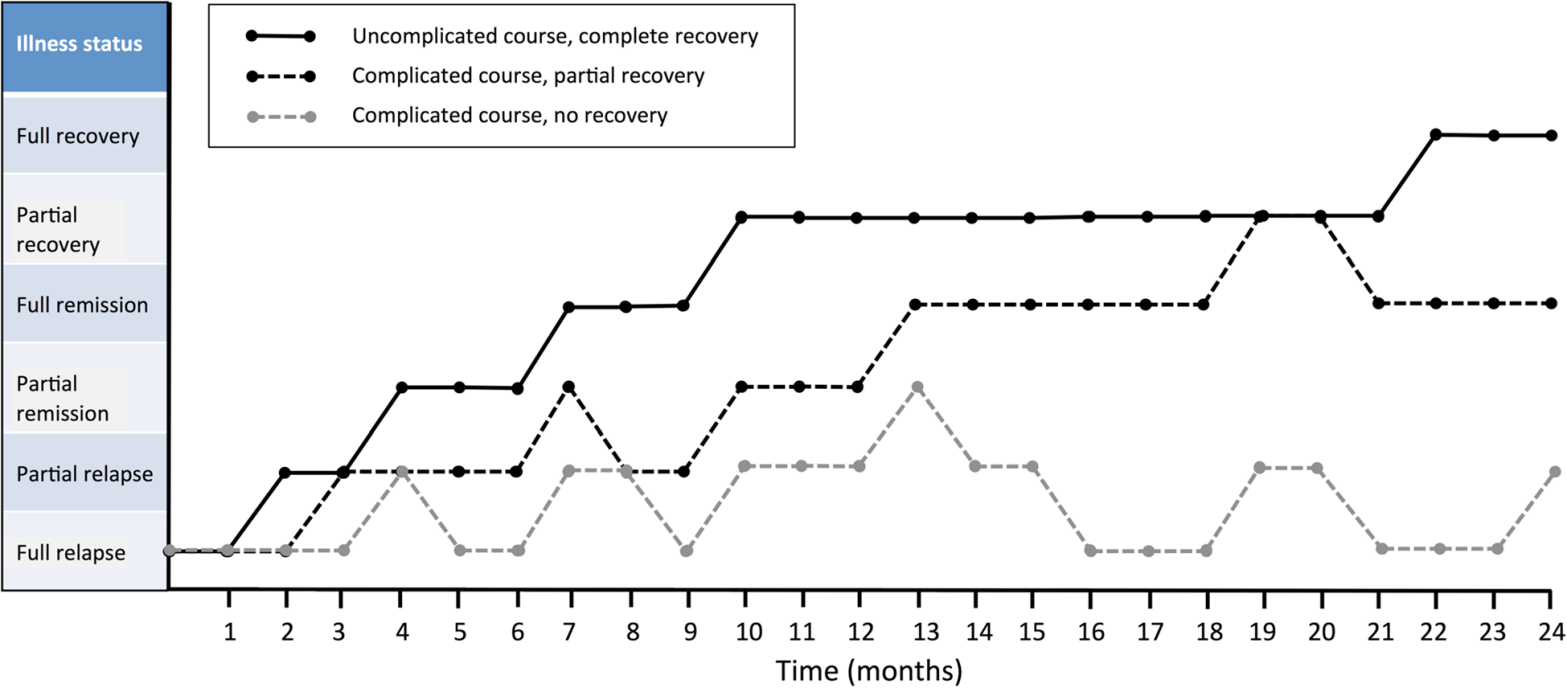
Illness trajectories across a 2 year time period for three hypothetical individuals with AN exhibiting different illness courses. One individual with an uncomplicated course shows a consistent transition from full relapse to full remission to full recovery. Another individual shows a complicated course marked by partial remission, partial relapse, and partial recovery, followed by a decline to full remission. A third individual shows a complicated course with no recovery marked by intermittent bouts of full relapse punctuated by partial relapse and partial remission. For an analogous depiction of illness trajectory based on actual patients, see Kordy et al., [10]

We propose a unifying framework with potential definitions for recovery, remission, and relapse to energize the discussion (see Fig. 2). These definitions are internally logical, consistent, and conducive to longitudinal assessment of AN. We advocate the adoption of standardized definitions for *partial* and *full* recovery and *partial* and *full* relapse. DSM-5 defines partial and full remission, but not partial or full recovery, and the duration requirement is vague (“a sustained period”) [41]. We propose that definitions of relapse in AN should encompass both clinical symptoms and signs such as BMI measures,^1^ as has been proposed for definitions of recovery [42], to more comprehensively capture the disorder. Importantly, our suggested criteria for recovery, remission, and relapse include objective measures (BMI; observable behaviors of restricting, binging, and purging), subjective measures (fear of gaining weight, disturbance of body image), standardized ratings (EDE), and specific durations of follow-up (1, 3, 6, and 12 months) that are conducive to utilization across both clinical and research settings (see Fig. 3).

It is worth noting that the proposed approach shares certain similarities with previous efforts to identify patterns of recovery in AN. For example, the Psychiatric Status Rating (PSR) scale represented a single six-item clinician rating based on DSM-III criteria [43]. Lower scores on this scale, such as a 1, indicated ‘usual self’ or the absence of meeting diagnostic criteria, whereas higher scores, such as a 6, indicated presence of ‘definite criteria, severe.’ The PSR is similar to our proposed approach in the sense that both require clinician ratings, and both load upon features of AN that are relevant to diagnostic criteria in terms of weight status, symptom burden, and ongoing behaviors. However, our proposed criteria diverge principally with respect to (1) a focus on defining stages of relapse, remission, and recovery, (2) reliance upon a standardized and clinically validated interview (EDE), and (3) utilization of terminology (partial or full relapse, partial or full remission, partial or full recovery) that are transparent and can be utilized uniformly with patients, caregivers, and clinicians. Our EDE cutoff selection for partial relapse (greater than or equal to 2 SD below normal) is also consistent with the ‘cutoff point a,’ which as previously suggested by Jacobsen et al. [44], represents a conservative and stringent approach to determining clinically significant changes.

Due to the highest risk of relapse being in the first year [4, 17, 20, 32, 33] and relapse often occurring as early as 3 months post-treatment [4, 7, 15, 32], we recommend that longitudinal studies conduct follow up assessments no less than every 3 months for the first year, and every 6 months thereafter for longer studies. Without standardized definitions, a refined understanding of the specific outcomes posed by putative risk factors, and guidance on measurement, we are in danger of adding more variability to this literature. Clinically, standardized definitions for relapse, remission and recovery, combined with consistent monitoring, would help provide consistent and relevant feedback to patients and family members regarding their level of risk.

There are several important limitations to consider when interpreting this review. There is an inherent difficulty identifying the true risk factors predicting AN relapse given the disparate definitions of relapse and recovery provided to date, potentially giving our review the appearance that it is challenged by a lack of synthesis. We argue that this challenge is precisely what future studies would overcome by adopting and adhering to one set of standards. Secondly, our interpretations are restricted to the somewhat obvious conclusions that AN is: (1) characterized by high relapse rates, (2) that relapse rates increase with follow-up lengths, and (3) there are few reliable predictors. While it seems nearly impossible to glean generalizations from such heterogeneous findings, this highlights the necessity for consensus and standardized definitions. It is important to emphasize that while the current review has focused on AN, based in part, on our own research efforts, we believe that similar consensus standards are needed for other eating disorders such as bulimia nervosa, binge eating disorder, and unspecified eating disorder. Although advancing such definitions are beyond the scope of our qualitative review, we hope that highlighting this disparity will provoke further discussion and progress. Finally, adding a meta-analytic approach could derive ‘quantitative data’ characterizing outcomes, but at this point, would not be additively informative given the aforementioned limitations. This approach would be useful for a future analysis of aggregated studies using uniform definitions.

### The value of reaching consensus

It will be important to carefully consider the value of reaching consensus on definitions of relapse, remission, and recovery, who will benefit, and how a consensus would be best achieved. It is hard to imagine a lasting consensus without the support of eating disorder organizations. These include organizations which are science-oriented (e.g., Eating Disorder Research Society (EDRS) [45], Academy for Eating Disorders (AED) [46] European Council on Eating Disorders (ECED) [47]), clinician-oriented (AED, National Eating Disorders Association (NEDA) [48], and International Association of Eating Disorders Professionals (IAEDP) [49]), and patient and caregiver-oriented (e.g., Families Empowered and Supporting Treatment of Eating Disorders (FEAST) [50], National Alliance on Mental Illness (NAMI) [51], AED, and NEDA).

It is also necessary to prospectively consider the potential challenges to achieving a consensus. In this regard, the highly interdisciplinary perspectives required in the research and treatment of eating disorders (pediatrics, family medicine, psychiatry, psychology, nutrition and dietetics, social work, licensed therapy and counseling, and nursing) results in complex and often diverging multifactorial models, which risks a fracturing of consensus regarding these conditions.

Concrete suggestions for harmonizing this discussion include (1) the development of conference symposia, (2) cross-organization workgroups or task forces, and (3) the generation of consensus statements focused on the topic. Other practical considerations include feasibility assessments. For example, follow up frequency will always be of concern, and conducting monthly, quarterly, and perhaps even bi-annual follow-ups requires resources that may be infeasible for certain research groups. We would argue that follow up assessment occurring at any frequency should use a standardized approach that is comparable to other laboratories. In-person assessments might be supplemented by phone interviews, and/or the remote collection of collateral information from family members, and we observed evidence of this pragmatic approach in the literature surveyed in this paper.

## Conclusion

The heterogeneity and severity of AN presentation poses challenges to understanding why relapse occurs, and how to prevent it. We posit that the eating disorders community will benefit from considering, testing, and adopting standardized definitions for relapse, remission, and recovery. To galvanize this movement, we have attempted to provide a unifying framework with internally logical and consistent definitions. This framework is conducive to longitudinal clinical and research assessment, not only for AN, but for bulimia nervosa, binge eating disorder, unspecified eating disorder, and other eating disorders. Without consensus, uncertainty and variability in the reported recovery, remission, and relapse rates will persist. Standardizing definitions in AN is a critical first step in identifying at-risk individuals, and can ultimately advance the development and evaluation of treatments for this life-threatening illness.

## Abbreviations

AN: Anorexia nervosa
BMI: Body mass index
BN: Bulimia nervosa
DSM: Diagnostic and statistical manual of mental disorders
EDE: Eating Disorder Examination
EDNOS: Eating disorder not otherwise specified
PSR: Psychiatric Status Rating

## Footnotes

1 Since weight and height normally increase until age 20 in pediatric and adolescent populations, age- and gender- adjusted BMI percentiles for determining expected body weight (EBW) are more appropriate in these subgroups (see Le Grange et al., [52]).
